# Spontaneous Resolution and Recurrence of Aseptic Splenic Abscesses in a Patient With Newly Diagnosed Crohn's Disease

**DOI:** 10.7759/cureus.75682

**Published:** 2024-12-13

**Authors:** Fatima I Hsayan, Mariam Naboulsi, Antoine Abou Rached

**Affiliations:** 1 Internal Medicine - Gastroenterology, Faculty of Medical Sciences, Lebanese University, Beirut, LBN; 2 Internal Medicine, Faculty of Medical Sciences, Lebanese University, Beirut, LBN

**Keywords:** aseptic abscess, crohn's disease, inflammatory bowel disease, nodule size, spleen abscess, steroid treatment

## Abstract

Aseptic splenic abscesses are rare in the early phases of Crohn's disease and are typically reported in patients with longstanding illness or uncontrolled symptoms despite medical treatment. We present a case of recurrent aseptic splenic abscesses in a young man newly diagnosed with Crohn's disease, whose illness remained well-controlled. This unique case raises questions regarding the spontaneous resolution of aseptic splenic abscesses without steroid therapy and their recurrence without acute Crohn's disease flare-ups.

## Introduction

Extraintestinal manifestations of Crohn's disease most commonly affect the joints, skin, and eyes, but other organs, such as the liver, pancreas, kidneys, and brain, can also be involved [1]. Splenic abscesses, though rare, are usually reported in later stages of Crohn's disease [2]. In the general population, septic splenic abscesses are often associated with infective endocarditis, with streptococcus and staphylococcus being the primary pathogens [3].

Aseptic splenic abscesses, on the other hand, are linked to noninfectious conditions, including systemic lupus erythematosus, rheumatoid arthritis, sarcoidosis, lymphoma, leukemia, metastatic solid tumors, and inflammatory bowel diseases (IBD). In most cases, these abscesses develop as late complications in patients with refractory disease or advanced illness stages [2]. Steroid therapy and biologic agents have been associated with the resolution of such abscesses [4].

Here, we report a rare case of multiple aseptic splenic abscesses that resolved spontaneously without steroid treatment in a newly diagnosed Crohn's disease patient, challenging the conventional understanding of their pathogenesis.

## Case presentation

An 18-year-old male with no prior medical history presented with a one-month history of fatigue and nonspecific abdominal pain. Initial investigations revealed: hemoglobin 11.9 g/dL (ref: 13-17.7 g/dL), mean corpuscular volume 69 fL (ref: 80-100 fL), iron 23 mcg/dL (ref: 59-158 mcg/dL), ferritin 48 ng/mL (ref: 25-400 ng/mL), elevated C-reactive protein (CRP) 29.3 mg/dL (ref: <3 mg/dL), and elevated fecal calprotectin 1012 mg/kg (positive: >200 mg/kg).

**Figure 1:**
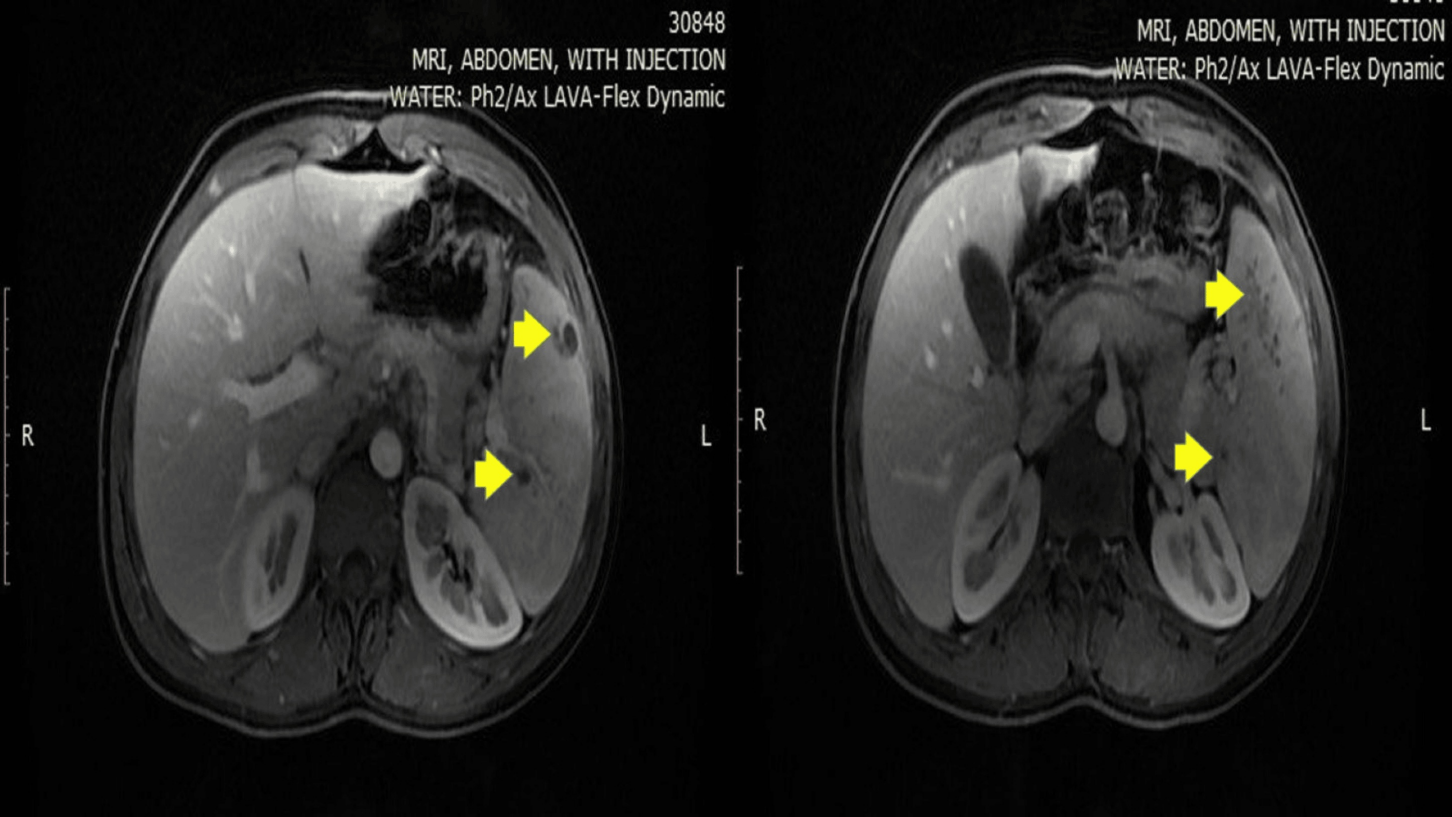
MRI abdomen with IV injection shows numerous ring enhancing lesions (yellow arrows) with imaging features, suggestive of splenic abscesses.

A colonoscopy revealed terminal ileitis consistent with Crohn's disease, and gastroscopy showed gastritis with granulomas on biopsy. The patient was initiated on budesonide (9 mg daily for one month, tapered to 6 mg daily for the second month). Two months into treatment, the patient presented with left upper quadrant pain but no fever. Laboratory findings included normal white blood cells and mildly elevated CRP. Imaging findings included splenomegaly with multiple hypodense splenic nodules on a contrast-enhanced CT scan, confirmed by MRI as splenic abscesses (Figure 1).

**Figure 2:**
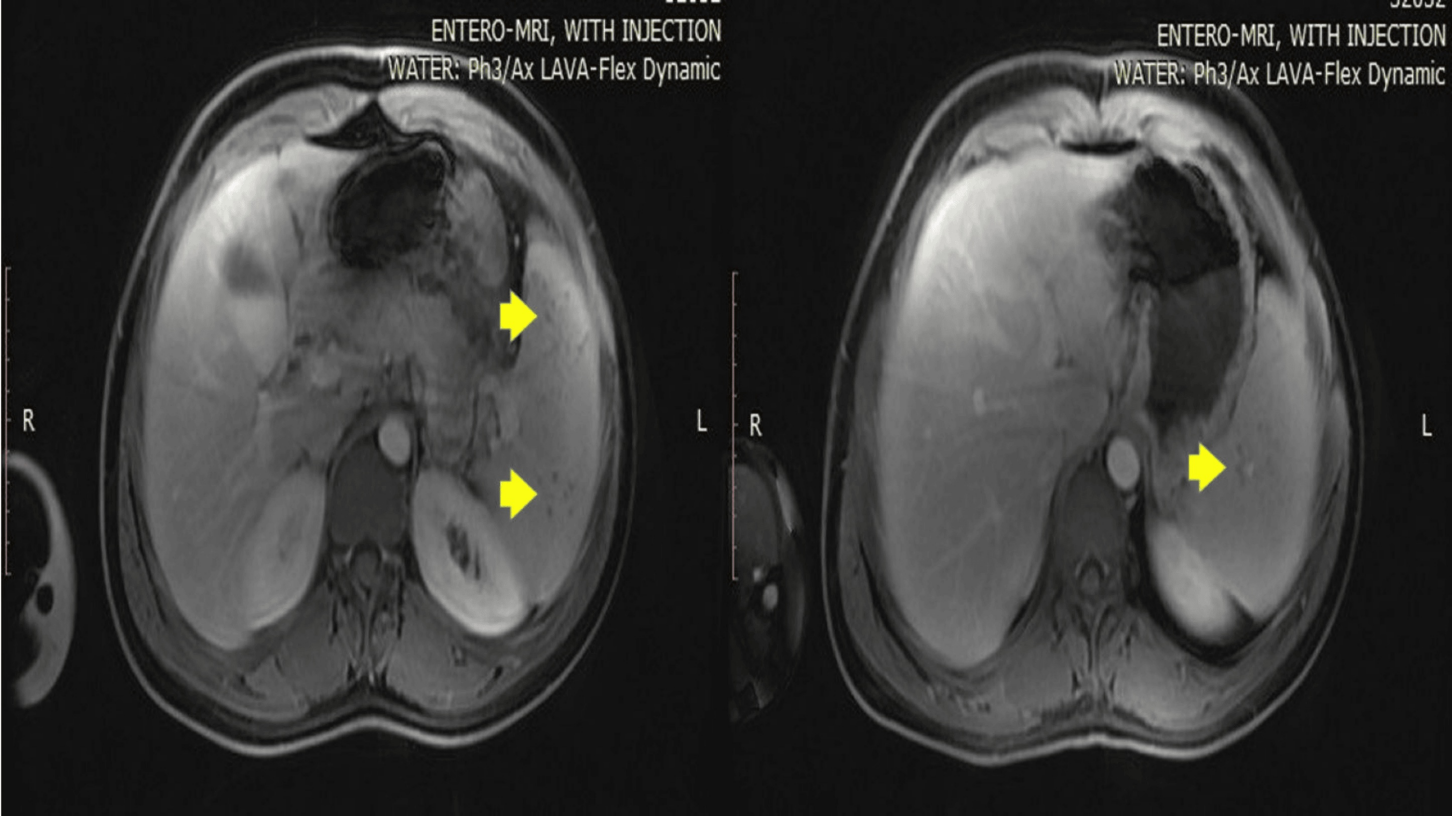
Magnetic resonance enterography shows an interval decrease in splenomegaly and hypointense lesions (yellow arrows) within the spleen. It is hypointense at present with no evidence of diffusion restriction and no evidence of ileitis.

Extensive investigations, including microbiological and autoimmune testing (blood and urine culture, stool cultures, interferon-gamma release assay (IGRA), toxoplasma IgG and IgM, brucella agglutination (febrile agglutinins), IgM and IgG Epstein-Barr virus serology, and cytomegalovirus IgM serology), were done and were found to be negative. Antibody tests for dsDNA and antinuclear antibodies (ANA) were also negative. The lesions were classified as aseptic splenic abscesses, likely an extraintestinal manifestation of Crohn's disease. The patient was discharged without additional treatment. Four months later, magnetic resonance enterography (MRE) showed a significant reduction in splenic lesions and splenomegaly, with no signs of active ileitis (Figure 2).

**Figure 3:**
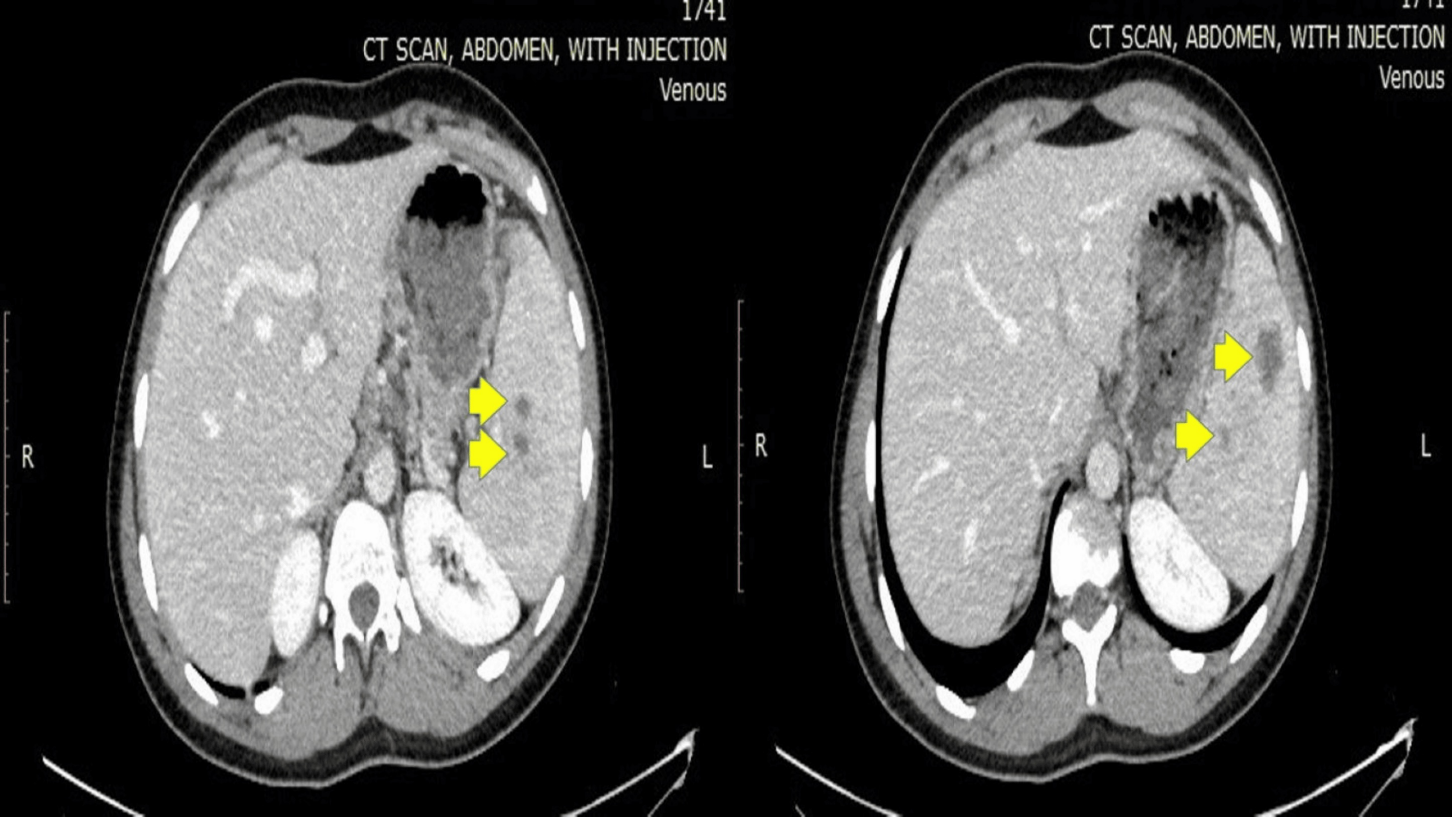
CT abdomen pelvis with IV injection shows an interval increase in the size of the hypo-enhancing splenic lesions (yellow arrows) that are likely related to the previously described splenic abscesses in the context of Crohn's disease, suggestive of an acute flare of Crohn's disease.

Ten months after the initial diagnosis, the patient presented again with left upper quadrant pain. Imaging revealed enlarged splenic lesions, corresponding to recurrent aseptic splenic abscesses (Figure 3), despite the absence of a clinical Crohn's flare. Colonoscopy showed mild ileitis, consistent with chronic inflammation. Steroid therapy was considered, and we followed up with the patient after treatment.

## Discussion

This case highlights an unusual presentation of aseptic splenic abscesses in a newly diagnosed Crohn's disease patient. While such abscesses are typically linked to active disease flares or advanced illness, our patient experienced spontaneous resolution without steroid therapy followed by recurrence without significant flare-ups.

Aseptic spleen abscesses are considered part of extraintestinal manifestations associated with IBD. Extraintestinal complications are experienced at least once by half of IBD patients, can be encountered even prior to diagnosis, and are more frequently noted in Crohn’s disease affecting the colon [5]. However, in our case, mild terminal ileitis was noted in the asymptomatic patient, two months before the appearance of an aseptic abscess.

Comparing our findings with similar cases, a 43-year-old woman with a history of Crohn's disease developed active colitis and was found to have splenic abscesses, which resolved only after treatment with high-dose steroids [6]. Also, a 24-year-old woman with splenic abscesses and Crohn’s symptoms had partial resolution with prednisolone but relapsed upon tapering [7]. Another patient, who was experiencing a Crohn’s flare with multiple splenic abscesses, improved after treatment with corticosteroids and biologics [4]. All of these cases showed resolution of the splenic abscess after steroid therapy. In contrast, our patient experienced spontaneous resolution of the splenic abscess without steroid therapy. Additionally, our patient had a recurrence of the aseptic abscess without a flare-up of Crohn’s disease, maintaining a stable disease course compared to the cases mentioned earlier, in which the patient experienced a Crohn’s flare-up with multiple splenic abscesses.

Similarly to our case, aseptic abscess syndrome (AAS) is a rare condition associated with systemic inflammatory disorders like IBD, which can affect patients with known Crohn's disease or ulcerative colitis or may precede the diagnosis. It most often involves the spleen and liver, but other potential sites of involvement include the lungs, lymph nodes, kidneys, pancreas, testes, and brain. The most common initial presentation includes fever, abdominal pain, weight loss, and peripheral leukocytosis [6]. However, our patient did not have systemic symptoms such as fever or weight loss. Corticosteroids and immunosuppressive therapies are effective in the majority of AAS cases [8]. Noting the difference in pathophysiology related to AAS, quantification of microbiota-associated metabolites and characterization of the T-regulatory/T-helper type 17 (Treg/Th17) cell balance will provide mechanistic insight into how the microbiota may be involved in the pathophysiology of AAS [9].

## Conclusions

This case underscores the need to recognize rare extraintestinal manifestations like aseptic splenic abscesses in Crohn’s disease, even in newly diagnosed patients. The spontaneous resolution and recurrence of splenic abscesses without active disease flares present a unique clinical challenge, warranting further investigation into their pathogenesis and management.
